# Antibiotic prescribing in primary care, adherence to guidelines and unnecessary prescribing - an Irish perspective

**DOI:** 10.1186/1471-2296-13-43

**Published:** 2012-05-28

**Authors:** Marion Murphy, Colin P Bradley, Stephen Byrne

**Affiliations:** 1 Department of General Practice, School of Medicine, University College Cork, Cork, Ireland; 2 Pharmaceutical Care Research Group, School of Pharmacy, University College Cork, Cork, Ireland

## Abstract

**Background:**

Information about antibiotic prescribing practice in primary care is not available for Ireland, unlike other European countries. The study aimed to ascertain the types of antibiotics and the corresponding conditions seen in primary care and whether general practitioners (GPs) felt that an antibiotic was necessary at the time of consultation. This information will be vital to inform future initiatives in prudent antibiotic prescribing in primary care.

**Methods:**

Participating GPs gathered data on all antibiotics prescribed by them in 100 consecutive patients’ consultations as well as data on the conditions being treated and whether they felt the antibiotic was necessary.

**Results:**

171 GPs collected data on 16,899 consultations. An antibiotic was prescribed at 20.16% of these consultations. The majority were prescribed for symptoms or diagnoses associated with the respiratory system; the highest rate of prescribing in these consultations were for patients aged 15–64 years (62.23%). There is a high rate of 2^nd^ and 3^rd^ line agents being used for common ailments such as otitis media and tonsillitis. Amoxicillin, which is recommended as 1^st^ line in most common infections, was twice as likely to be prescribed if the prescription was for deferred used or deemed unnecessary by the GP.

**Conclusion:**

The study demonstrates that potentially inappropriate prescribing is occurring in the adult population and the high rate of broad-spectrum antimicrobial agents is a major concern. This study also indicates that amoxicillin may be being used for its placebo effect rather than specifically for treatment of a definite bacterial infection.

## Background

Antibiotic resistance is a major concern globally. Antibiotic consumption increases the likelihood for an individual to develop bacterial resistance [1]. The majority of antibiotic prescribing takes place in primary care and general practitioners (GPs) have been encouraged to prescribe antibiotics more rationally and to only give antibiotics when necessary [2].

Wide variation in antibiotic prescribing practices has been shown to exist in Europe demonstrating more than a threefold difference in antibiotic prescribing rates between countries, without any logical reasoning to explain the variation [3].

In the Republic of Ireland (ROI), there has been increasing levels of resistance and antibiotic use [4]. Quinolone resistance rates in *E.coli* increased from 5% in 2002 to 23% in 2008. Although ROI has seen small reductions in antibiotic use since 2009, high seasonal variation is still apparent which, in other countries, is also associated with high antibiotic consumption (France, Greece, Portugal and Italy) [3]. The reductions in antibiotic use seen in ROI have been associated with the stabilisation of rates of penicillin non-susceptible *Streptococcus Pneumoniae* (PNSP). The proportion of PNSP increased in ROI from 10.3% in 2004 to 23.1% in 2008 and decreased in 2010 to 18.2% [5]. The reductions in antibiotic use in ROI is low in comparison to that of Belgium and France where sustained campaigns have been associated with reductions of 36% and 26.5% respectively [6,7].

Non-clinical factors such as patient pressure have been shown to influence prescribing even in the absence of a clinical indication for an antibiotic [8,9]. The majority of ROI citizens must pay a fee to visit their GP and this has shown as a possible influence on the GP’s decision to prescribe an antibiotic during the consultation [10].

The purpose of this study was to describe specific diagnoses for which systemic antibiotics are prescribed in primary care by GPs in the ROI, to assess adherence to recent national guidelines and gain more information on antibiotics that are prescribed unnecessarily.

## Methods

### Study population

The majority of GPs in ROI (> 80%) are regular attendees of the small group continuing medical education (CME) network which is run by the Irish College of General Practitioners (ICGP). The CME network is provided in a format whereby GPs meet on a regular basis to learn in a mutually supportive group. The system is resourced by 37 CME tutors nationally with a target population of 1,934 GPs and an attendance of over 80% of all GPs in Ireland [11] Tutors run monthly meetings each year from September to May.

All CME tutors nationally and their CME groups were invited to participate in the study from October 2008 to April 2010. Each GP also completed an anonymous demographic questionnaire detailing their practice size, area, years in practice and post-graduate experience. All participating GPs were both state and privately funded. The state issues General Medical Service (GMS) cards to patients that are deemed unable to pay for medical care. Eligibility for GMS cards is dependent on a number of factors including income, marital status and age. In 2009, 33% of the population in Ireland were GMS card holders [12]. All other patients (private patients) must pay for their medical care.

### Procedure

Participating GPs gathered data on 100 consecutive consultations using a predefined piloted data collection proforma (Additional file 1). Out of hours consultations were not recorded. No data was collected during the summer months. Anonymised patient information was recorded including the age, gender and patient payment status. GPs recorded the reason for the consultation, or the diagnosis (if a diagnosis was reached). These were classified by the associated body system e.g. respiratory, skin, urinary tract disorders. All consultations associated with the respiratory system were further categorised into clinical entities and classified as symptoms (e.g. sore ear) or as diagnoses (e.g. otitis media). These categories are listed in Table 1. When an antibiotic was prescribed during the consultation, details of the prescription and directions for use were recorded on the data collection proforma provided. GPs were also asked to record if they felt the antibiotic was necessary, not necessary or unsure. It was also recorded if the patient had received an antibiotic for the same condition in the previous two weeks. Each antibiotic prescribed and the reason for the consultation was compared to locally approved guidelines on community prescribing of antibiotics in Ireland [13]. These guidelines contain up-to-date evidence on the use of antibiotics for common conditions that present in primary care e.g. tonsillitis, otitis media. There were four types of deviations detected based on: (1) choice of antibiotic; (2) duration/frequency of antibiotic; (3) dosage of antibiotic; (4) diagnosis. Deviations due to diagnosis were recorded when antibiotics were prescribed for conditions not included in the guidelines, due to the lack of evidence to support their use (e.g. sore throat, cough). GPs were encouraged to provide additional information regarding the antibiotic prescription particularly if it was an influence on their decision (e.g. pregnancy, sensitivity analysis) and this was taken into account when deviations were assessed.

**Table 1:** Symptoms and diagnoses associated with the respiratory system

**Clinical Entity**	**Symptoms**	**Diagnosis**
**Ear**	Ear symptoms	Otitis media Otitis externa
**Upper Respiratory Tract**	Coryza/Rhinitis Nasal congestion Head cold Post nasal drip Sneezing	Upper respiratory tract infection
**Throat**	Sore throat Throat symptoms	Tonsillitis Pharyngitis Trachetis Peritonsula/Quinsy Laryngitis Croup
**Sinus**	Sinus pain/symptoms	Sinusitis
**Lower Respiratory Tract**	Dyspnoea Cough Cough & sputum/phlegm Wheezing Pleural effusion Pleuritic Pain	Community acquired pneumonia Lower respiratory tract infection Bronchitis
**Asthma/COPD**		Asthma COPD Exacerbation
**Other**	Pyrexia/Fever/Temperature Unspecified respiratory tract infection	Cystic Fibrosis Bronchiolitis/RSV Bronchiectasis

### National antibiotic consumption data

Data regarding antibiotic consumption in ROI during the time of the study (October 2008-April 2010) was provided by the Health Protection Surveillance Centre (HPSC). This data is from IMS Health, a pharmaceutical market research company and contains monthly wholesaler to retail pharmacy sales data from over 95% of the wholesalers and manufacturers in Ireland. Antibiotic consumption was measured in Defined Daily Dose (DDD) which is the assumed average maintenance dose per day for a drug used for its main indication in adults.

### Analysis

Data was analysed using Microsoft Office Excel® (2007) and Predictive Analytics SoftWare (PASW®, Chicago, Illinois, USA) version 17.0. A sample (10%) was double-checked for coding entry errors. Data collected was tested for normality and parametric/non-parametric tests were used as appropriate. The Pearson’s chi-squared tests were performed to compare categorical variables. Odds ratios (ORs) with corresponding 95% confidence intervals (CIs) were calculated using logistic regression.

HPSC data was used to compare the pattern of antibiotic usage between the national picture and the study GP population.

### Ethical approval and Informed Consent

The study was approved by the Clinical Research Ethics Committee of the Cork Teaching Hospitals. Informed consent was obtained from all GPs by participation in the study.

## Results

Data were collected from 171 GPs nationally who recorded information on 16,899 consultations. The mean (±SD) number of consultations recorded per GP was 98.82 ± 5.37. The majority of GPs who participated completed a demographics questionnaire (84.80%, 145). All GPs were from combined GMS and private practices and situated in various settings: urban (37.24%, 54), rural (24.83%, 36) and mixed (37.93%, 55)

The majority of attendees were females (58.80%, 9,936) and held a GMS card (53.83%, 9,096).

An antibiotic was prescribed in one-fifth of consultations recorded (20.16%, 3,407). A quarter of these (25.74%, 877) were prescribed to a child between the ages of 1 and 14 years. A small proportion had received an antibiotic for the same presenting complaint in the previous two weeks prior to the consultation (8.22%, 280).

### Choice of antibiotic

Co-amoxiclav, amoxicillin and clarithromycin were the most commonly prescribed antibiotics and this was also reflected in the national data (Table 2). With the exception of doxycycline and lymecycline, the pattern of antibiotic usage in terms of ranking was very similar.

**Table 2:** Comparison of the most common antibiotics consumed nationally in primary care and the most common antibiotics prescribed by General Practitioners in the study. (DDD: defined daily dose; HPSC: Health Protection Surveillance Centre)

**Antibiotic**	**National consumption data (HPSC)**			**GP Study**		
	**Rank**	**DDD**	**%**	**Rank**	**Reported prescriptions**	**%**
Co-amoxiclav	1	765,235	27.01	1	792	23.66
Amoxicillin	2	448,362	15.83	2	719	21.48
Clarithromycin	3	386,060	13.63	3	308	9.20
Doxycycline	4	176,445	6.23	9	81	2.42
Flucloxacillin	5	128,998	4.55	5	231	6.90
Phenoxymethylpenicillin	6	113,971	4.02	4	233	6.96
Cefaclor	7	108,434	3.83	6	173	5.17
Trimethoprim	8	107,384	3.79	7	131	3.91
Lymecycline	9	97,918	3.46	19	20	0.60
Erythromycin	10	89,771	3.17	8	82	2.45
Ciprofloxacin	12	84,981	3.00	10	78	2.33
Other antibacterials		325,666	11.49		500	14.93

### Reasons for an antibiotic prescription

Symptoms/diagnoses associated with the respiratory system accounted for the majority of antibiotic prescriptions (64.72%, 2,205), followed by skin (10.21%, 348) and urinary tract disorders (8.63%, 294). Overall, 22.63% of consultations (3,824) recorded either a diagnosis or symptoms of the respiratory system. The majority of these consultations received an antibiotic prescription (57.66%, 2,205). Children aged 4–14 years had the highest consultation rate where a respiratory symptom/diagnosis was recorded (33.94%, 1,298). Patients under the age of 65 were twice as likely to consult with respiratory symptoms/diagnoses than younger patients (OR 2.08, 95% CI 1.87-2.23).

**Figure 1:**
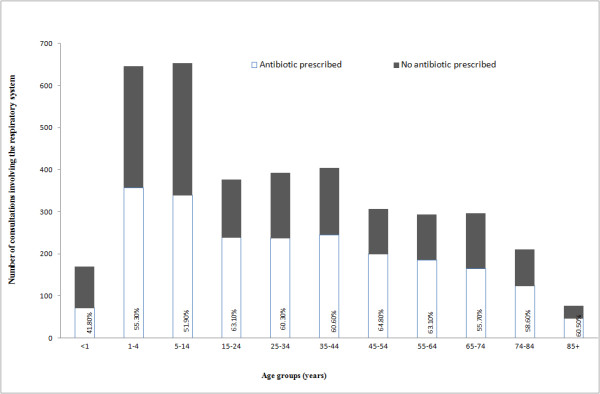
The number of consultations involving respiratory symptoms/diagnoses by age group and the percentage of antibiotics prescribed.

**Figure 2:**
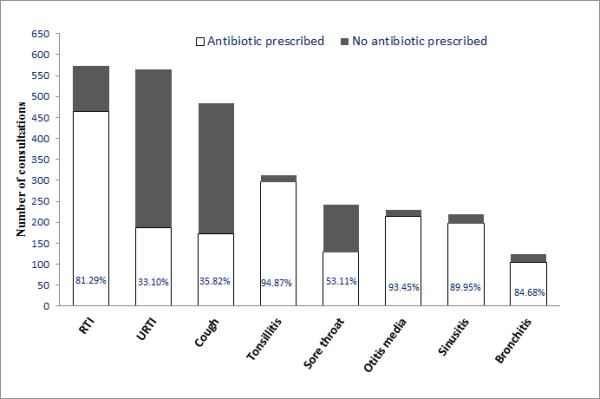
Common respiratory conditions and the percentage of antibiotic prescribing for each condition.

Children aged from 0–14 years had the lowest percentage rate of antibiotic prescribing when presenting with respiratory symptoms (52.25%, 767). The highest percentage was seen for patients aged 15–64 (62.23%, 1,104) (Figure 1). In consultations with a respiratory diagnosis, 66.08% (1,420) resulted in an antibiotic prescription, in comparison with 48.22% (787) of those with respiratory symptoms. High prescribing rates (>85%) were seen in patients with throat, ear and sinus infections recorded. There was also considerable antibiotic prescribing for conditions not included in the guidelines such as upper respiratory tract infection (URTI) (33.10%, 187), cough (35.82%, 173) and sore throat (53.11%, 128) (Figure 2).

### Adherence to guidelines

The majority of antibiotic prescriptions were not strictly in accordance to guidelines (78.05%, 2,659). Nearly half of the deviations were related to the diagnosis/reason given for the consultation (46.71%, 1,242). This correlated with the high use of antibiotics for respiratory symptoms which are not included in the guidelines. The choice of the antibiotic was also a recurrent deviation (30.27%, 805). These included deviations such as the use of 2^nd^ and 3^rd^ line agents for infections as opposed to the 1^st^ line choice. Antibiotic prescriptions for common respiratory infections seen in the community corresponded with the recommended first-line choice in less than 50% of all prescriptions (Table 3). Co-amoxiclav accounted for almost a quarter of the prescriptions in tonsillitis (25.09%, 74), otitis media (25.46%, 84), bronchitis (27.75%, 187), sinusitis (23.71%, 46) and urinary tract infections (21.82%, 48).

**Table 3:** Percentages of first-line, second-line and remaining antibiotics according to Irish national guidelines

**Guidelines**	**First line (%)**	**Second line/remaining (%)**
Tonsillitis (n = 295)	Phenoxypenicillin (42.71)	Clarithromycin* (5.76) Amoxicillin (19.66) Co-amoxiclav (25.09) Other (6.78)
Otitis media (n = 330)	Amoxicillin (42.12)	Clarithromycin* (19.70) Co-amoxiclav (25.46) Cephalosporins (6.97) Other (5.46)
Sinusitis (n = 194)	Amoxicillin (15.46) Doxycycline (24.23)	Clarithromycin* (18.56) Co-amoxiclav (23.71) Cephalosporins (13.40) Other (4.64)
Bronchitis/Cough (n = 674)	Amoxicillin (36.50) Doxycycline (0.00)	Clarithromycin* (21.22) Co-amoxiclav (27.75) Cephalosporins (10.68) Other (3.86)
Community acquired pneumonia (n = 25)	Amoxicillin (0.00) Clarithromycin (44.00) Doxycycline (0.00)	Co-amoxiclav (44.00) Cephalosporins (4.00) Other (8.00)
Urinary Tract infection (uncomplicated female) (n = 220)	Trimethoprim (32.73) Nitrofurantoin (7.73)	Cephalosporins (20.00) Co-amoxiclav (21.82) Quinolones (10.91) Other (6.82)

*Clarithromycin/Erythromycin is first line in penicillin allergy.

The majority of antibiotic prescriptions were for immediate use (84.00%, 2,862); co-amoxiclav (23.72%, 679), amoxicillin (18.20%, 521) and clarithromycin (9.54%, 273) were the most common antibiotics prescribed. There were 470 (13.80%) antibiotic prescriptions for deferred use (2.05% (70) were not recorded). The majority of these prescriptions were amoxicillin (39.57%, 186), followed by co-amoxiclav (19.57%, 92) and phenoxymethylpenicillin (9.15%, 43). When a deferred prescription was issued, it was twice as likely to be for amoxicillin as any other antibiotic (OR 2.18, 95% CI 1.78-2.66).

### Necessary antibiotics

**Figure 3:**
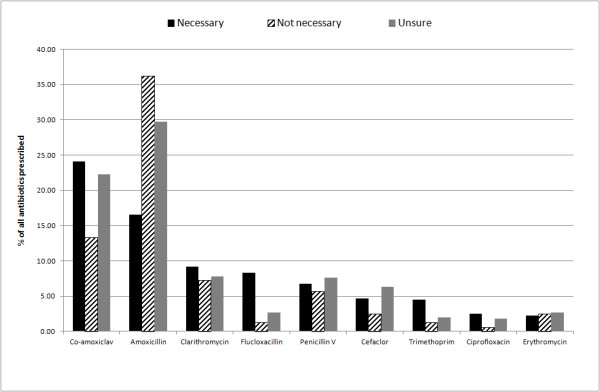
The percentage of the most common antibiotics prescribed that were deemed necessary (n = 2,445), not necessary (n = 315) and unsure (n = 602) for the presenting patient.

Most of the antibiotics that were prescribed were deemed to be necessary for the condition they were being prescribed for (71.76%, 2,445) and almost a quarter of these prescriptions were for co-amoxiclav (24.09%, 589). There were 315 (9.25%) prescriptions that were thought not to be necessary and prescribers were unsure of 602 (17.67%) prescriptions; amoxicillin was the most common antibiotic choice in these instances (36.19%, 114 and 29.73%, 179 respectively) (Figure 3). Amoxicillin was twice as likely to be prescribed when the antibiotic was thought to be unnecessary (OR 2.86, 95% CI 2.22-3.69). URTI was the most common reason for an unnecessary prescription (17.46%, 55) followed by cough (12.06%, 38) and flu-like symptoms (9.52%, 30).

## Discussion

Overall, this study has identified the huge burden of respiratory symptoms/conditions in general practice in Ireland. Nearly a quarter of all the consultations recorded were due to respiratory illnesses (22.63%). This is higher than the UK where only approximately 15% of GP consultations are due to these ailments [14]. As with other countries, the most common cause for an antibiotic prescription is due to symptoms or conditions relating to the respiratory system. Overall, children (1–14 years) received the highest proportion of the antibiotic prescriptions and this is probably due to the high number of consultations involving the respiratory system in this age group. Surprisingly, patients (15–64 years) presenting to the GP have the highest rate of antibiotic use per respiratory consultation, with over 60% of these consultations receiving an antibiotic prescription. Other studies have also shown that children visit their GP more often with respiratory conditions but in proportion do not receive more antibiotics than adults [14,15]. Research has also shown that antibiotics are not justified to reduce the risk of serious complications of these conditions but may be warranted for those over the age of 65 where the number needed to treat to prevent pneumonia post chest infection is much lower than younger age groups (39 vs 119) [16]. Therefore it would be expected to see higher rates of antibiotic use in the elderly cohort. Possible explanations for the higher rates of antibiotic use seen in younger patients (15–64 years) may be due to structure of the health care system in ROI. The elderly, the very young (<5 years) and the most socio-economically disadvantaged are more likely to be GMS card holders due the eligibility criteria. The average cost for a GP consultation for a private patient in ROI is €51 [17]. GPs have been shown to over-estimate the patient’s expectation for a prescription and this influences the decision to prescribe [18]. This may be more evident in ROI as GPs may be more sensitive to patients’ expectations if the patient is paying for the consultation [10]. It may be particularly difficult for the GP not to give a prescription under these circumstances; they may provide a prescription to reduce the need for re-consultation if required. Diagnostic uncertainty and fear of negligence have also been quoted as drivers of antibiotic overprescribing [19].

There is considerable variation in antibiotic prescribing between the respiratory clinical entities with a substantial proportion of prescribing occurring for respiratory symptoms. GPs may have had an inclination to label symptoms as a diagnosis to gain endorsement of their prescribing; diagnostic labelling has been shown to be associated with high levels of antibiotic prescribing [20].

Antibiotics are not recommended for symptoms such as cough, sore throat but a high level of prescribing was recorded in these conditions. This is the reason why the majority of deviations from guidelines in this study were due to the diagnosis/reason for consultation. A study in Canada showed that 5% of URTI consultations received an antibiotic; this is in contrast to 33.10% in this study [21]. The majority of consultations that diagnosed an ear infection received an antibiotic (89.49%); USA and the UK have similar rates while the Netherlands has much lower rates of 56% [22,23]. In a recent study in the USA, 30% of children diagnosed with a viral respiratory infection were prescribed an antibiotic [24].

In comparison to other countries, the level of agreement to guidelines is low. However, these guidelines have been recently developed and should be disseminated widely to have any impact. In the Netherlands, about three-quarters of antibiotics prescribed are first-line choice [25]. A third of patients who had been prescribed an antibiotic in the previous two weeks (i.e. for the same illness) had been prescribed co-amoxiclav which is recommended as second-line (broad-spectrum antibiotic); this could suggest that these cases were not treatment failures but rather viral illnesses.

The majority of tonsillitis presentations are not being treated with narrow-spectrum antibiotics as considered appropriate. This has a major impact on increasing resistance. The usage of amoxicillin for otitis media (first line) is lower than expected at 42.42%, other countries in Europe have rates of 82% with co-amoxiclav only used in 7% of cases [23]. Studies have described how a reduction in antibiotic usage carries a parallel decrease in resistance [26,27]. The high incidence of co-amoxiclav has been shown in other studies in ROI, with the consumption increasing by 80% from 2000–2005 while the use of amoxicillin decreased by 9% in the same time period [28,29]. In the absence of guidance from public health bodies, it could be argued that third-party representatives from the pharmaceutical industry may have contributed to the high usage of second line and third line agents [30].

In contrast to the high rates of co-amoxiclav, amoxicillin was more likely to be prescribed than other antibiotics when the prescription was for deferred/delayed use and also when the GP did not think that the antibiotic was necessary for the condition being treated. It could be argued that this study provides possible evidence that GPs prescribe antibiotics such as amoxicillin as ‘impure placebos’ or ‘pseudoplacebos’, these agents consist of biologically active agents that have specific efficacy for some conditions but used as a placebo for another condition i.e. antibiotics for probable viral infections such as URTI and cough. A survey in Denmark found that 70% of GPs had prescribed antibiotics as a placebo intervention in the previous year [31]. The most common reason quoted for this was to ‘*follow the wish of the patient and avoid conflict*’ [31]. Prescribers also report generally positive attitudes about the use of medication to promote the placebo effect [32].

This study clearly indicates that guidelines need to be disseminated at a local level with a focus to reduce antibiotic prescribing for minor respiratory conditions and reduce broad-spectrum antibiotic prescribing whenever necessary. Prescribing a course of antibiotics increases the patient’s risk of developing bacterial resistance for up to a year [1]; GPs should be mindful of this issue when prescribing antibiotics that are not necessary. It is particularly important to increase public awareness regarding appropriate antibiotic use to ease patient pressure on GPs to prescribe.

### Strengths and weaknesses

The data from this study is valuable as it is the first time diagnoses are linked to antibiotic consumption in ROI and also details prescribing behaviour for both private and GMS card holders. In ROI, private patients are not required to register with their GP and are therefore free to visit any GP they wish; patients do not have a unique identifier. Therefore, it is currently not possible for routine data collection to take place from electronic sources in ROI.

The reason for the consultation was recorded in free text words to include all types of reasons provided for an antibiotic prescription. Other studies have quoted that a large proportion of primary care visits are difficult to label with a precise code and also require GPs to participate in training in coding [14]. Therefore in this study, we were unable to differentiate between multiple consultations pertaining to the same episode. However, the number of previous antibiotics for the same episode was low.

The population of GPs in this study may be skewed towards those with an interest in the area of antibiotic prescribing as their participation was not mandatory as part of their CME group. However, the national data from the time of study reflects similar usage patterns of antibiotics. A possible explanation for the notable higher usage of doxycycline and lymecycline in the national dataset could be explained by dermatologists prescribing these agents in the outpatient setting.

## Conclusion

This study provides evidence that antibiotics are probably not being used as prudently in the community in ROI as they may be other countries. Children present to their GP with respiratory symptoms more often than adults, but higher rates of antibiotic prescribing occur in adults with similar symptoms. This study also shows that amoxicillin may be being used for its placebo effect rather than as specific treatment for bacterial infection.

## Competing interests

The authors declare that they have no conflict of interest.

## Authors' contributions

MM distributed the data collection booklets, analysed the data and drafted the original manuscript. All authors critically reviewed the manuscript and approved publication submission.

## Pre-publication history

The pre-publication history for this paper can be accessed here:

http://www.biomedcentral.com/1471-2296/13/43/prepub

## Supplementary Material

Additional file 1 **Data Collection form.pdf.** Description of data: This is a copy of the data collection form which participating GPs completed for the purposes of this study. Click here for file
