# Supplementary material for: Antibiotic prescribing in primary care, adherence to guidelines and unnecessary prescribing - an Irish perspective
Source: BMC Fam Pract. 2012 May 28;13:43. doi: 10.1186/1471-2296-13-43 (PMC3430589; doi:10.1186/1471-2296-13-43)
Supplement: Additional file 1 — Data Collection form.pdf. Description of data: This is a copy of the data collection form which participating GPs completed for the purposes of this study. [file 1471-2296-13-43-S1.pdf]

Antibiotic Review - Data Collection Form

Please complete using a black biro. Mark Boxes like this ☒ and write only in spaces provided.

Section 1

Q1. Date of Consultation ☐☐ / ☐☐ / ☐☐☐☐ *(e.g. DD/MM/YYYY)*

Q2. Age ☐☐ years/ months **or Date of Birth** ☐☐ / ☐☐ / ☐☐☐☐

Q3. Gender ☐ Male ☐ Female **Q4. GMS** ☐ Yes ☐ No

Q5. Reason for Encounter/Diagnosis

Q6. Was an antibiotic prescribed at this consultation? ☐ Yes ☐ No
(If Yes continue to Section 2)

Section 2

Q7. Name of antibiotic prescribed (if known allergy please state)

Q8. Dose Information

a. Times per day ☐☐ (e.g. three times daily = 03)

b. ☐☐☐☐ mgs (e.g. 1000mgs) **OR** ☐☐ . ☐☐ mls (e.g. 01.25 mls)

c. For how many days ☐☐ *(e.g. 7 days = 07)*

Q9. For immediate or deferred use? ☐ Immediate Use ☐ Deferred Use

Q9a. Do you believe this antibiotic was definitely necessary?
☐ Yes ☐ No ☐ Not Sure

Q9b. If no or not sure, please explain

Q10. Was another antibiotic recently prescribed for the same condition?
(<2 weeks ago, if Yes provide details below) ☐ Yes ☐ No

Section 3 - OFFICE USE ONLY - NOT TO BE COMPLETED BY GP

Q11. Compliant with guidelines? ☐ Yes ☐ No

Q12. Deviation from guidelines - specify ☐☐☐
